# Supplementary material for: A comprehensive benchmarking with practical guidelines for cellular deconvolution of spatial transcriptomics
Source: Nat Commun. 2023 Mar 21;14:1548. doi: 10.1038/s41467-023-37168-7 (PMC10027878; doi:10.1038/s41467-023-37168-7)
Supplement: Supplementary file 1 — Supplementary Information [file 41467_2023_37168_MOESM1_ESM.pdf]

A comprehensive benchmarking with practical guidelines for cellular deconvolution of spatial transcriptomics

Haoyang Li[1,2,†], Juexiao Zhou[1,2,†], Zhongxiao Li[1,2], Siyuan Chen[1,2], Xingyu Liao[1,2], Bin Zhang[1,2], Ruochi Zhang[3], Yu Wang[3], Shiwei Sun[4,5], Xin Gao[1,2]

[1]Computational Bioscience Research Center, King Abdullah University of Science and Technology (KAUST), Thuwal, Saudi Arabia

[2]Computer, Electrical and Mathematical Sciences and Engineering Division, King Abdullah University of Science and Technology (KAUST), Thuwal, Saudi Arabia

[3]Syneron Technology, Guangzhou, 510000, China

[4]Key Lab of Intelligent Information Processing, Institute of Computing Technology, Chinese Academy of Sciences, Beijing, 100190, China

[5]University of Chinese Academy of Sciences, Beijing 100049, China

Correspondence should be addressed to X.G. (email: xin.gao@kaust.edu.sa)

†These authors contributed equally to this work.

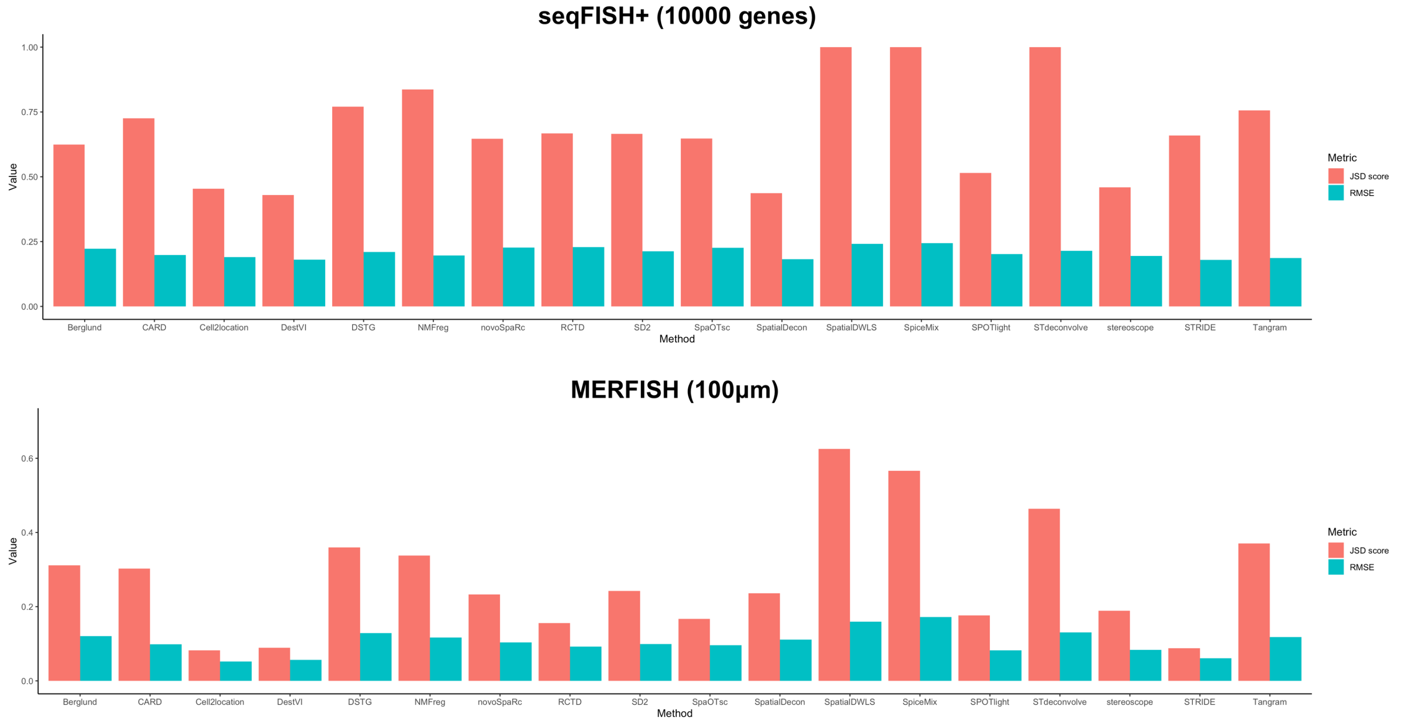

Supplementary Figure 1

The JSD and RMSE of two datasets (seqFISH+ with 10000 genes per spot and MERFISH under resolution of 100 μm) thourgh all methods. Source data are provided as a Source Data file.

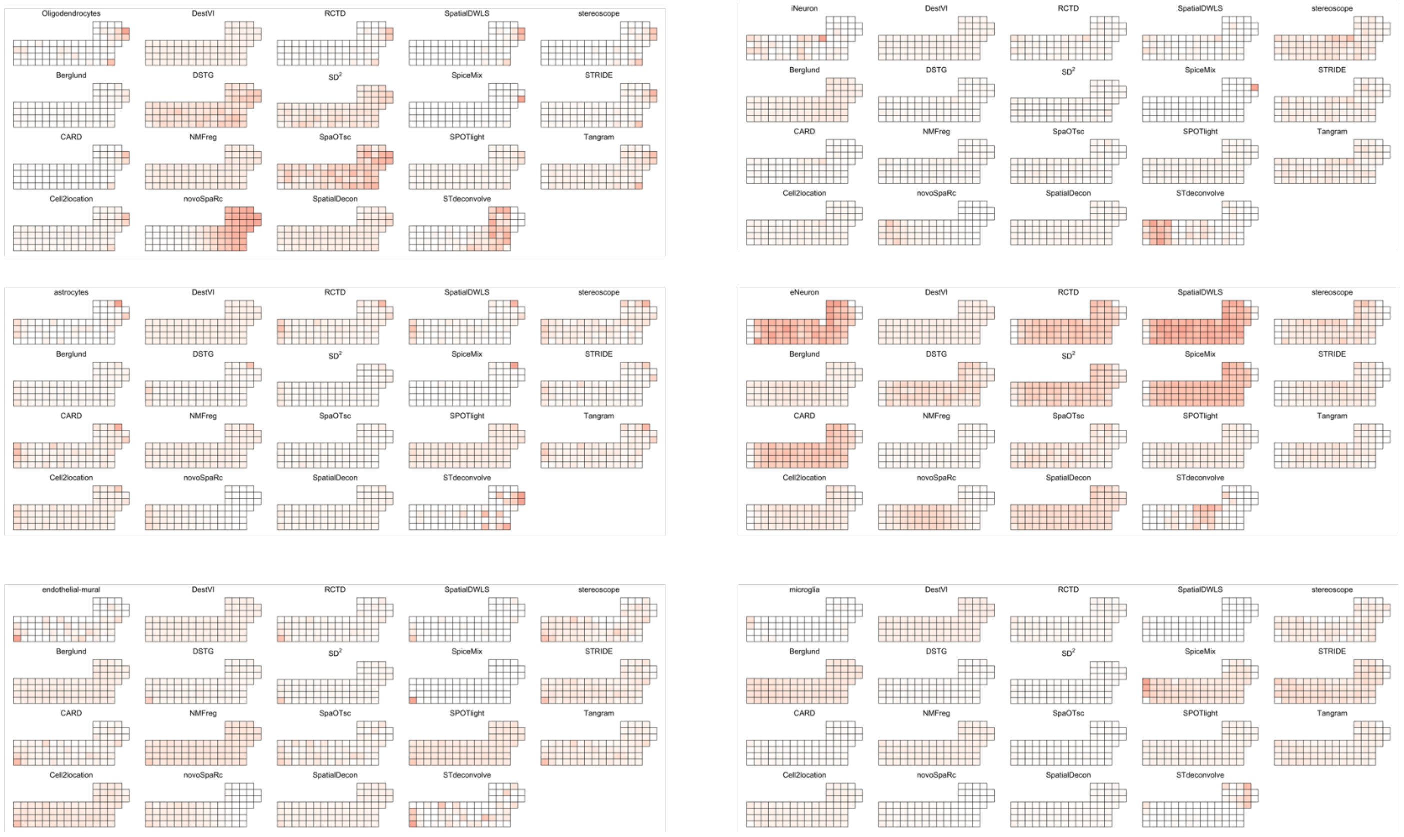

Supplementary Figure 2

The deconvolution results of six cell types through all methods in seqFISH+ dataset with 10000 genes per spot.

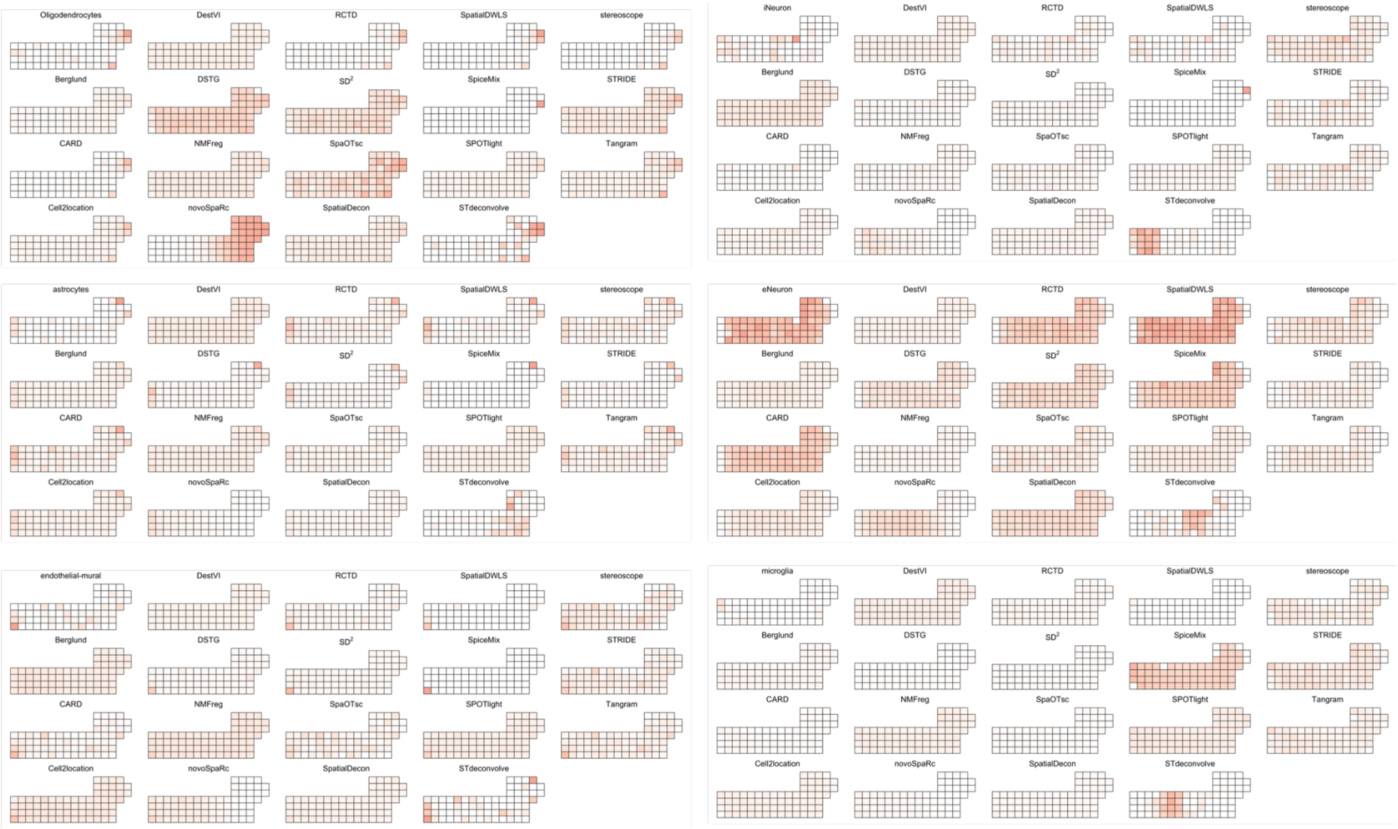

Supplementary Figure 3

The deconvolution results of six cell types through all methods in seqFISH+ dataset with 6000 genes per spot.

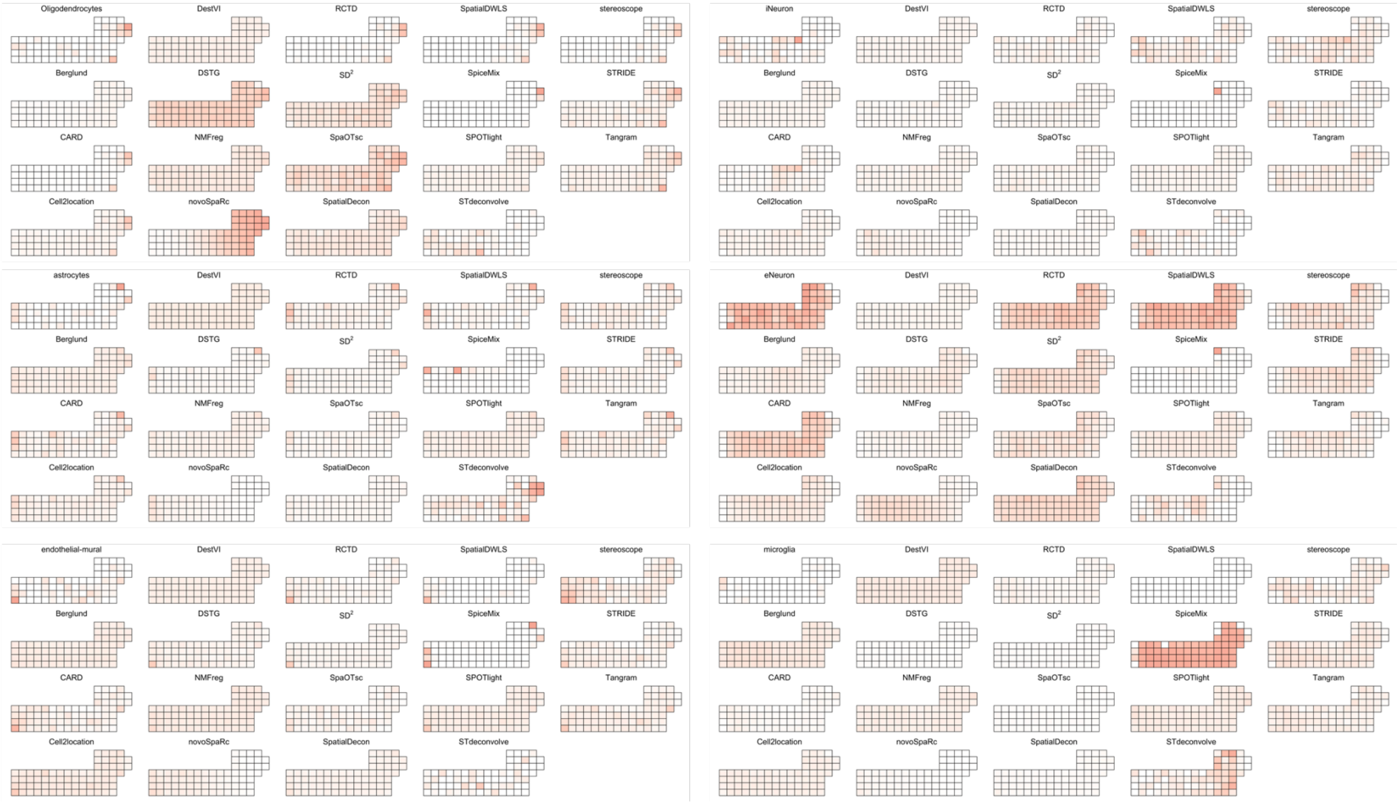

Supplementary Figure 4

The deconvolution results of six cell types through all methods in seqFISH+ dataset with 3000 genes per spot.

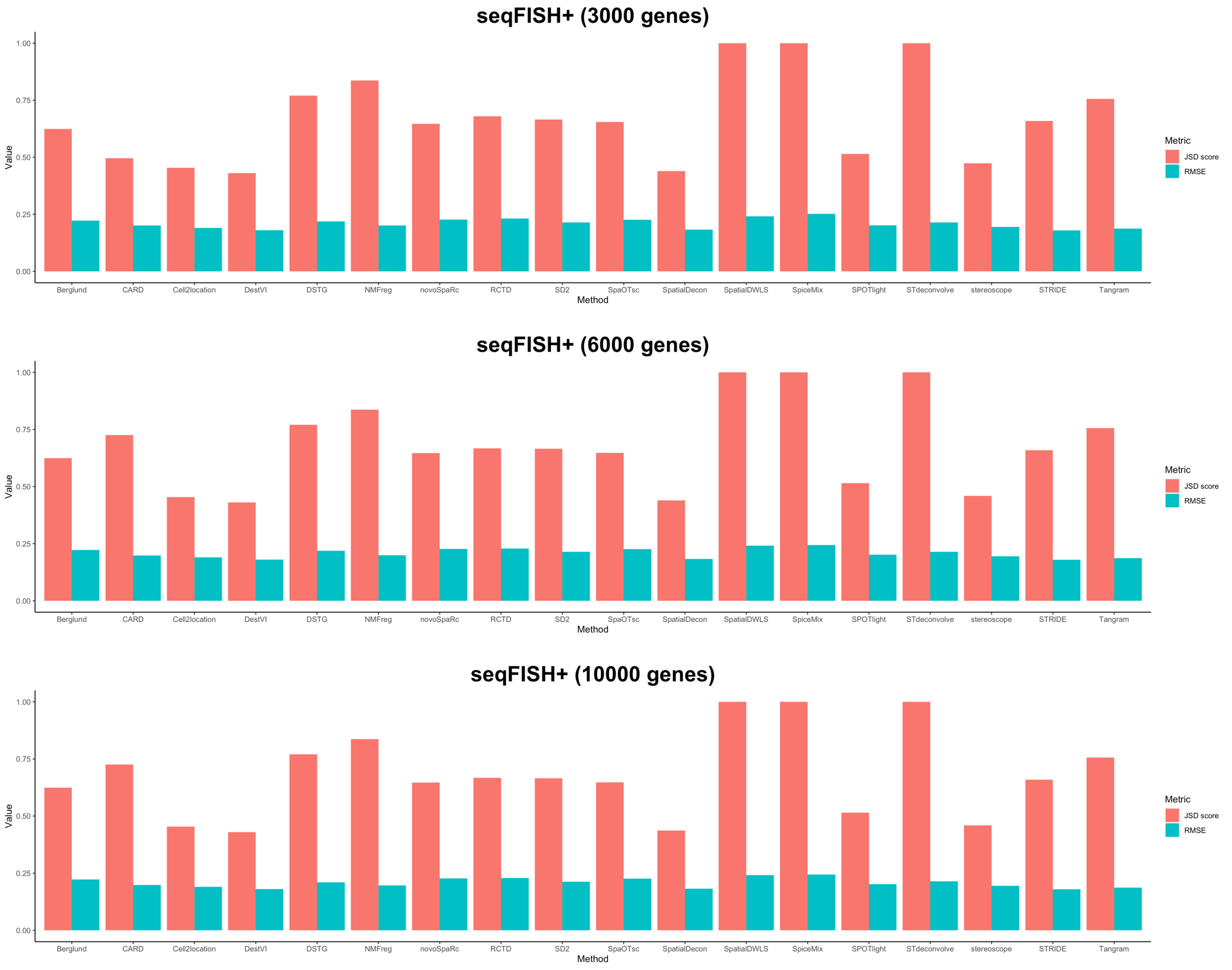

Supplementary Figure 5

The JSD and RMSE results of all cell types through all methods in seqFISH+ dataset with three kinds of gene number (10000, 6000, 3000) per spot. Source data are provided as a Source Data file.

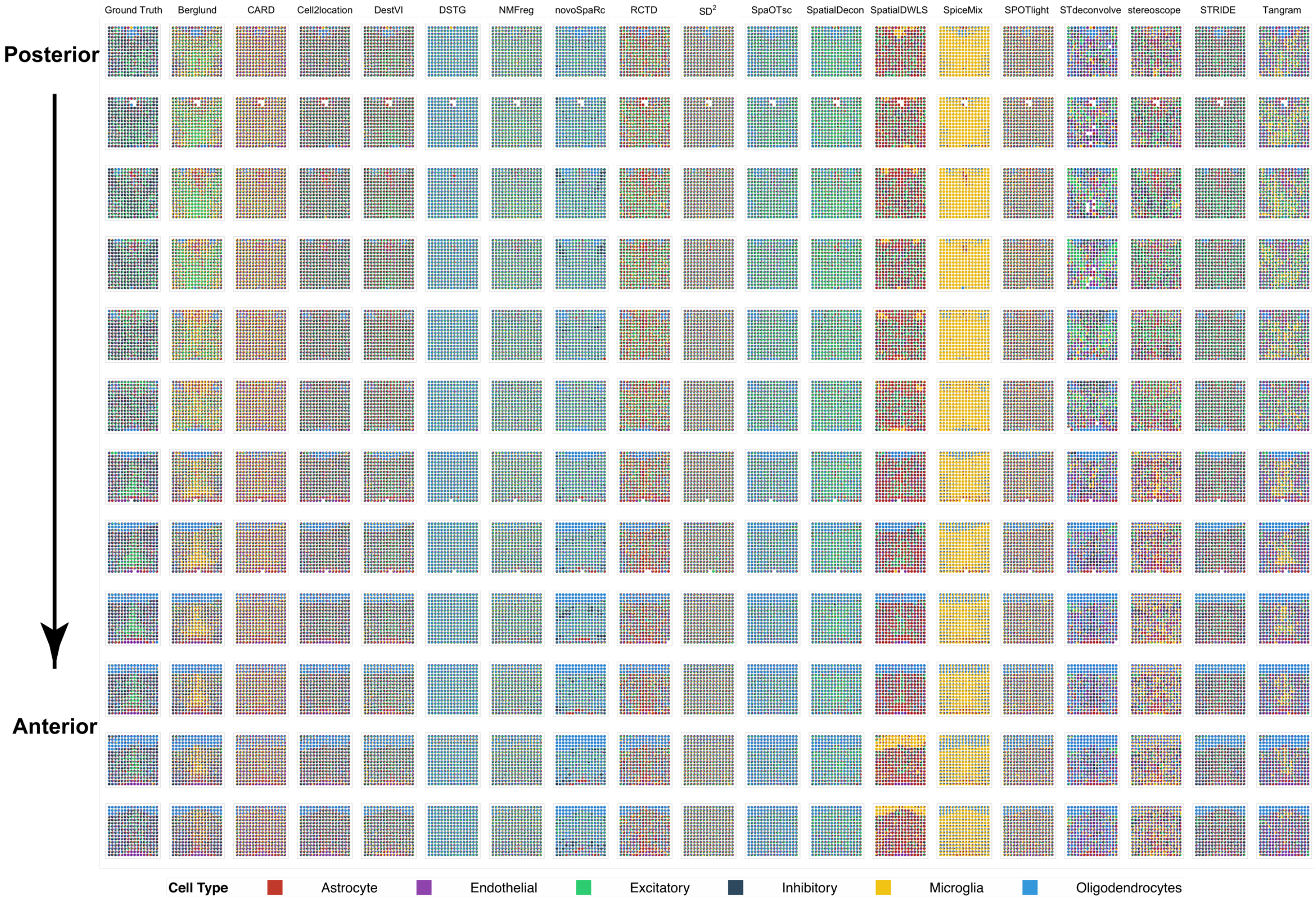

Supplementary Figure 6

The visualization results of all six cell types through all methods in MERFISH datasets with 100 μm resolution per spot. All 12 samples of MERFISH datasets from posterior to anterior are shown. The first column is ground truth.

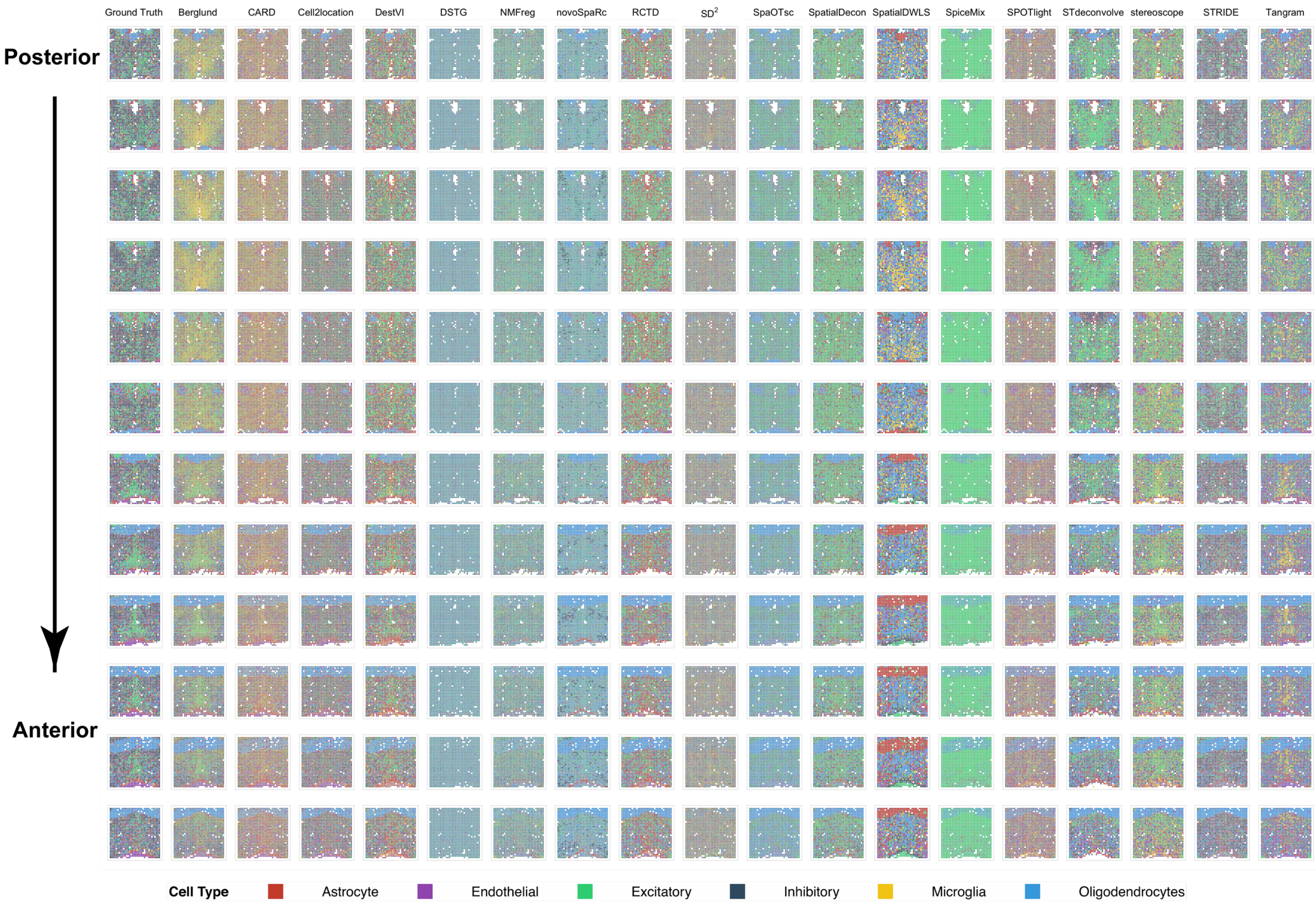

Supplementary Figure 7

The visualization results of all six cell types through all methods in MERFISH datasets with 50 μm resolution per spot. All 12 samples of MERFISH datasets from posterior to anterior are shown. The first column is ground truth.

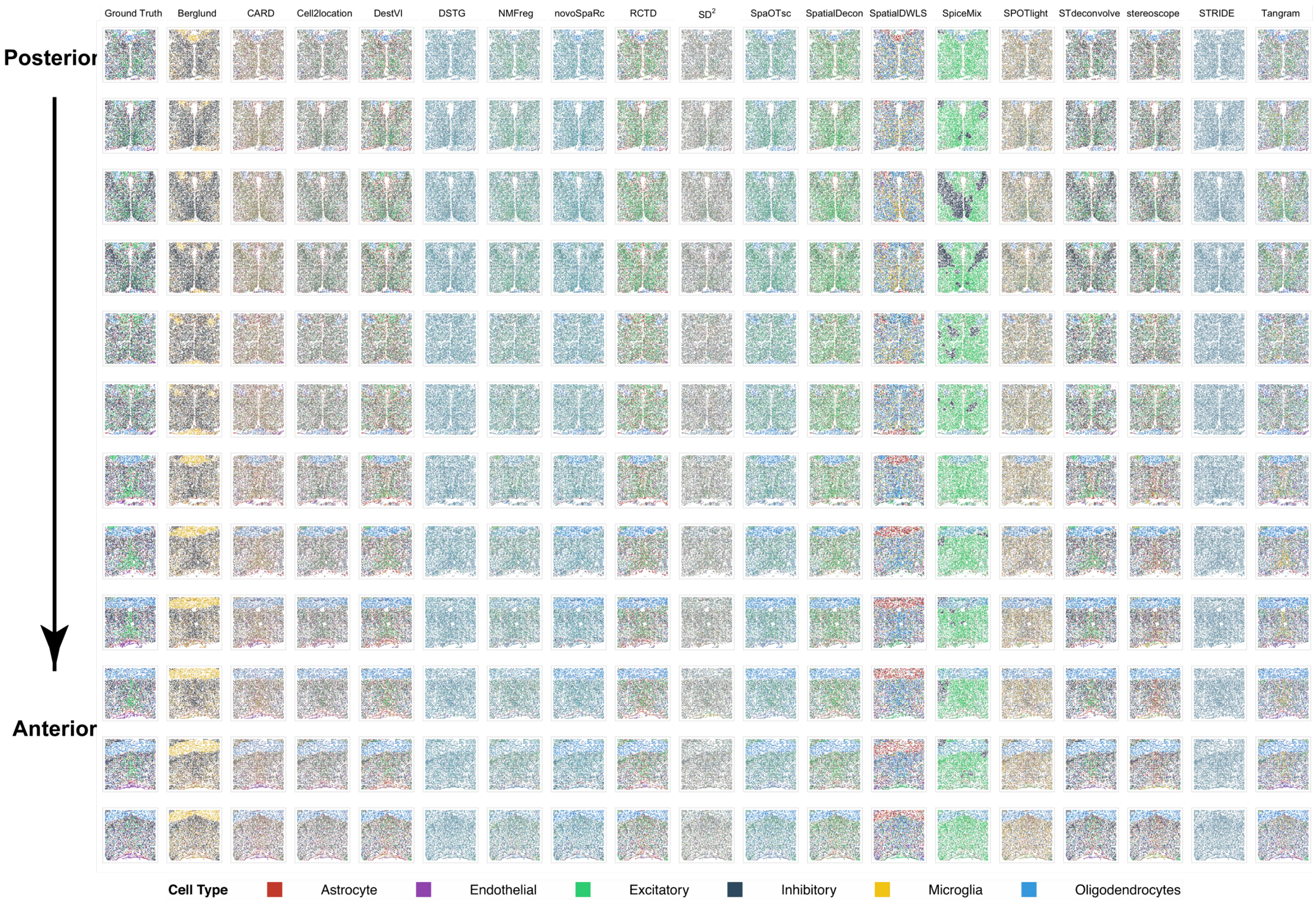

Supplementary Figure 8

The visualization results of all six cell types through all methods in MERFISH datasets with 20 μm resolution per spot. All 12 samples of MERFISH datasets from posterior to anterior are shown. The first column is ground truth.

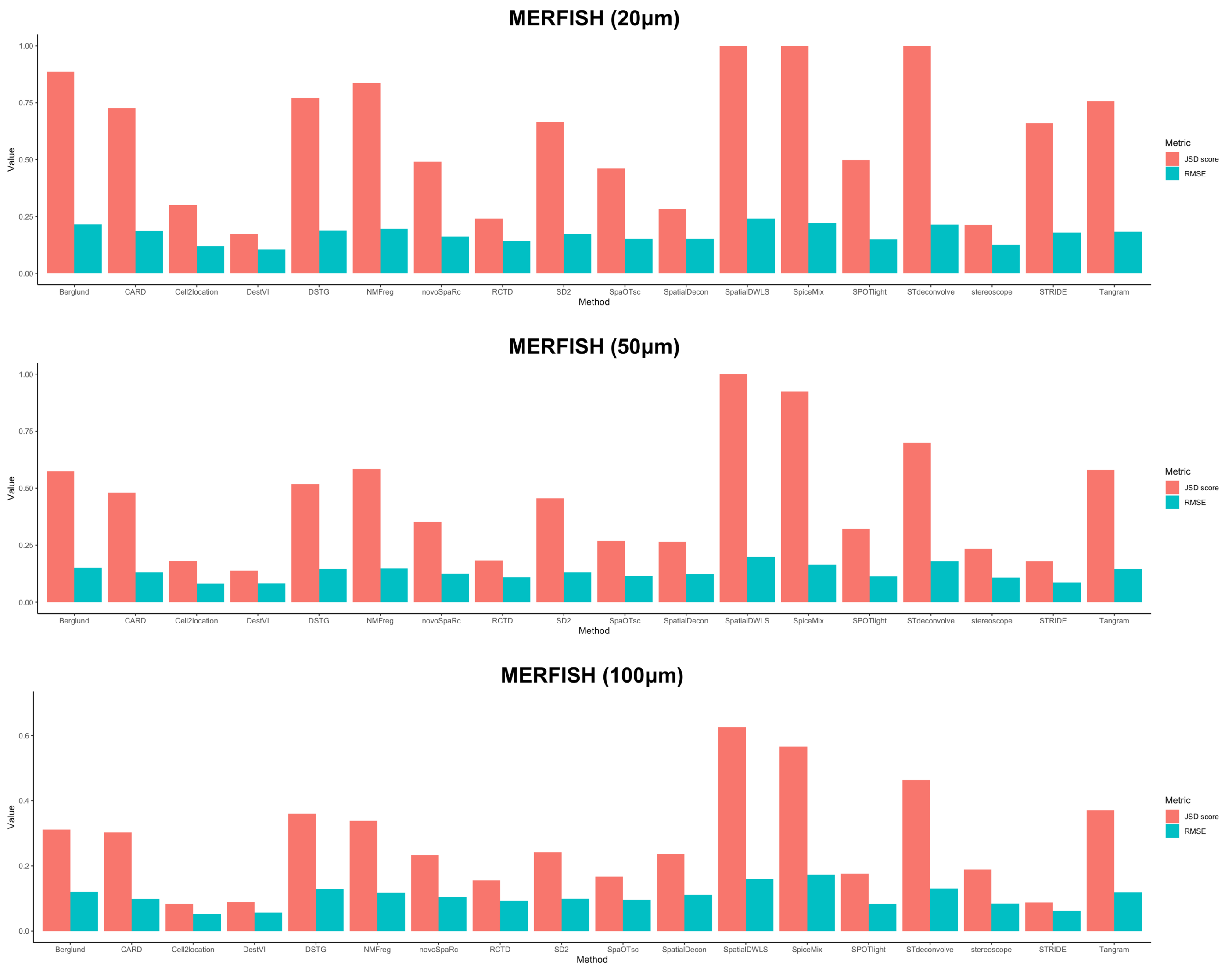

Supplementary Figure 9

The JSD and RMSE results of all cell types through all methods in MERFISH dataset with three kinds of resolution (100 μm, 50 μm, 20 μm) per spot. The shown JSD and RMSE are calculated by the mean of them through 12 samples and all cell types. Source data are provided as a Source Data file.

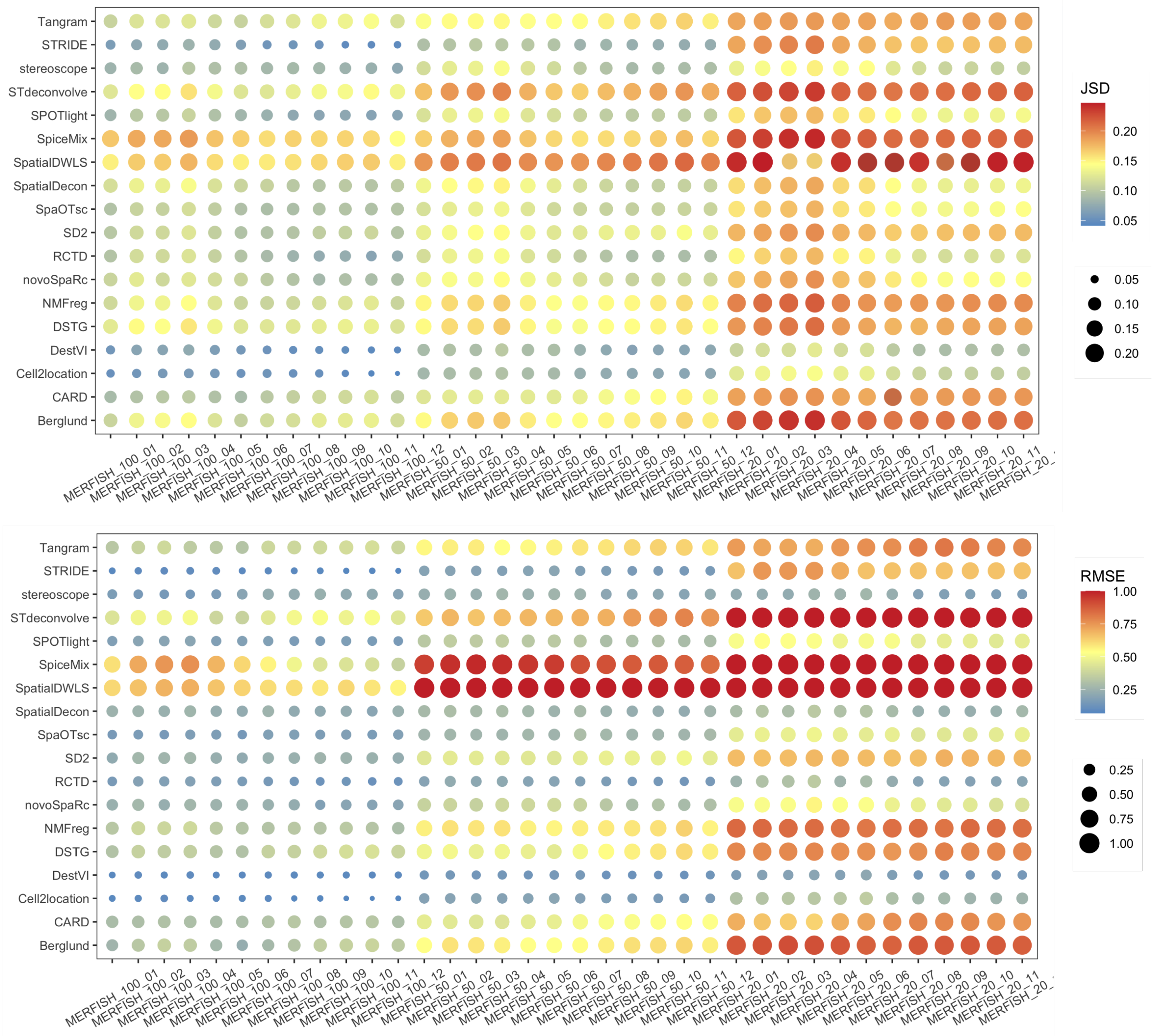

Supplementary Figure 10

The JSD (top) and RMSE (bottom) results of all cell types through all methods of all 36 samples in MERFISH datasets under three kinds of resolution (100 μm, 50 μm, 20 μm) and 12 samples. The shown JSD and RMSE are calculated by the mean of them through all cell types. The larger and darker dots represent the higher RMSE or JSD. Source data are provided as a Source Data file.

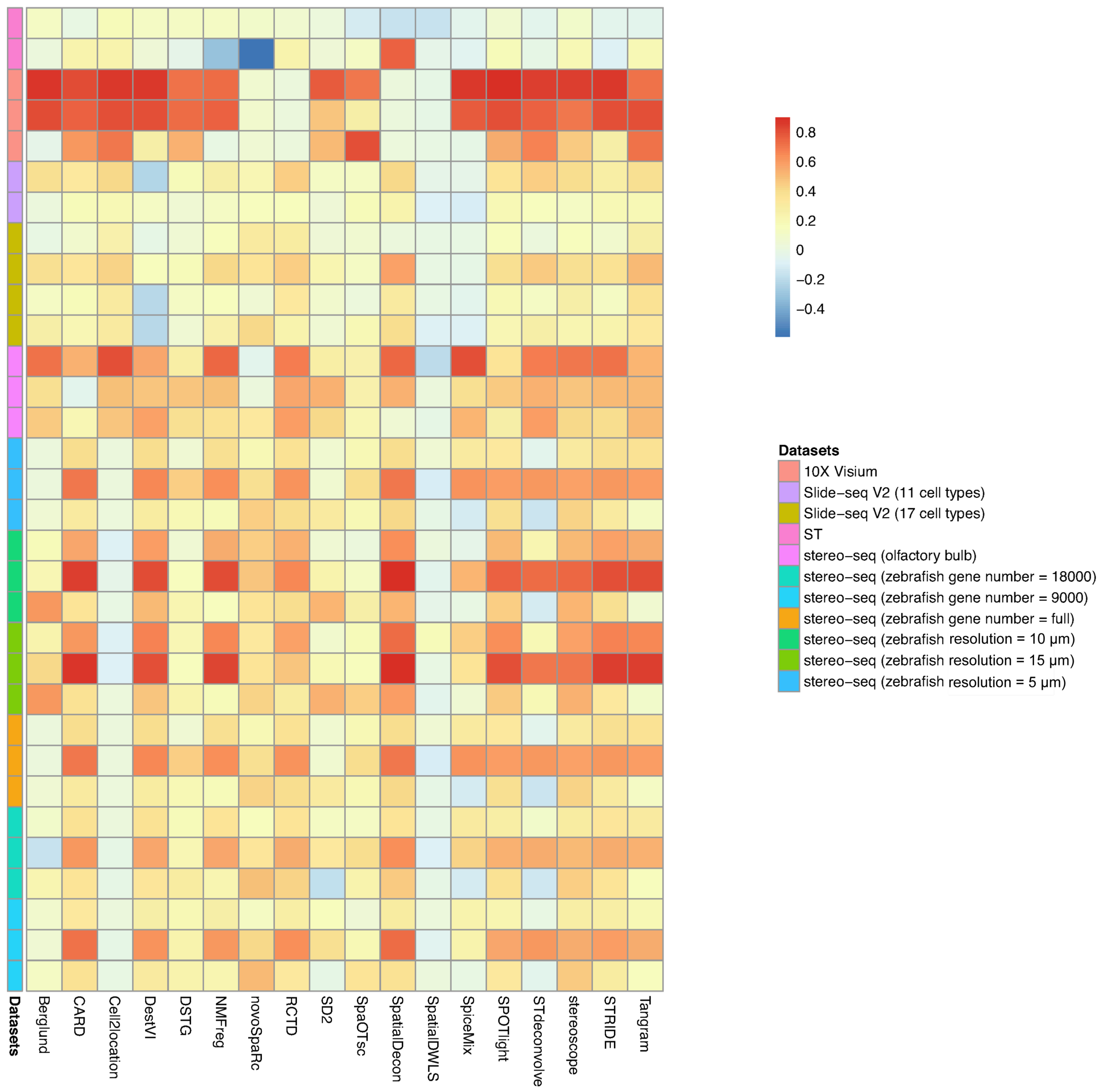

Supplementary Figure 11

The PCC heatmap for all pairs of predicted proportions of cell types and their expressions of marker genes through all methods. Each dataset is labeled by its specific color. In the heatmap, red represents high correlation and blue represents low correlation. Especially, the PCC of the third row of Visium by NMFreg is NaN, which resulted from the all zero proportion of that cell type (excitatory neurons of claustrum) NMFreg predicted. Source data are provided as a Source Data file.

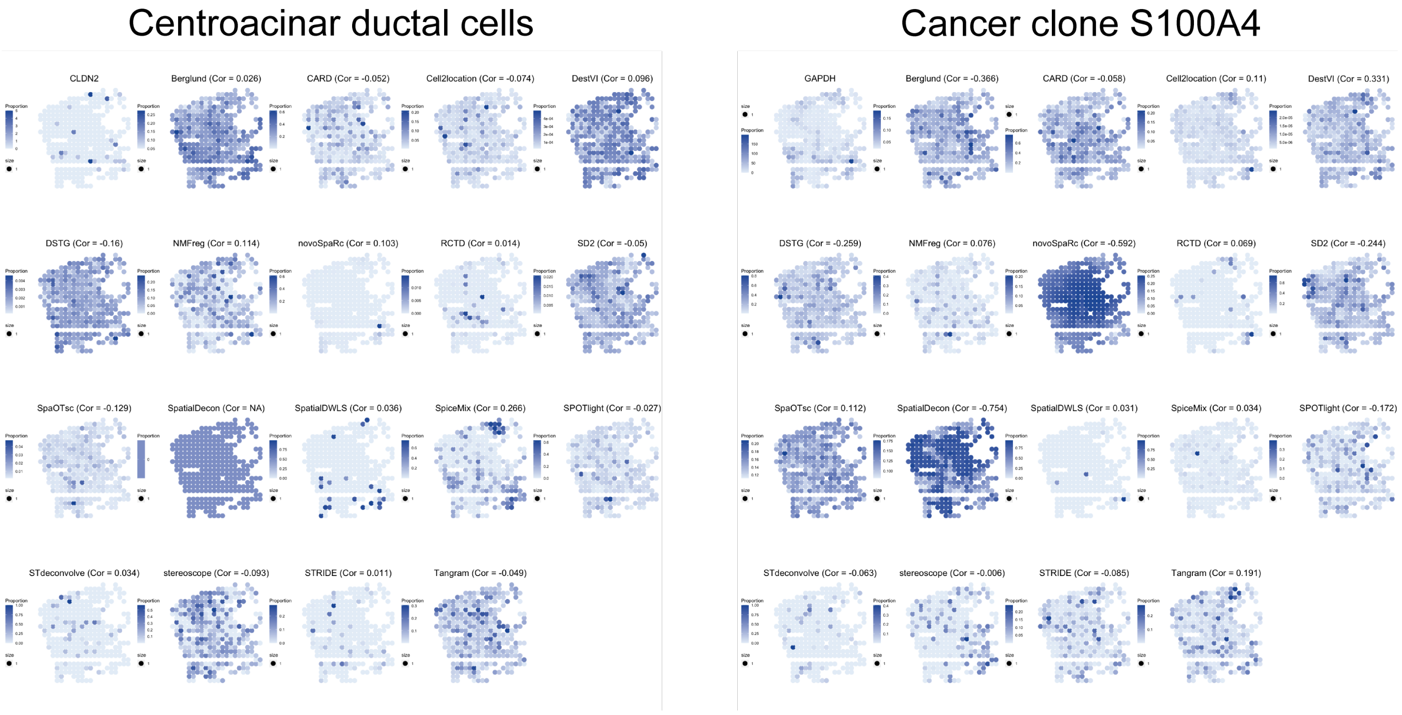

Supplementary Figure 12

The visualized prediction of two kinds of cell types (centroacinar ductal cells and cancer clone S100A4) through all methods and visualized spatial expression of marker genes (ground truth). Each method is labeled by its PCC value with ground truth.

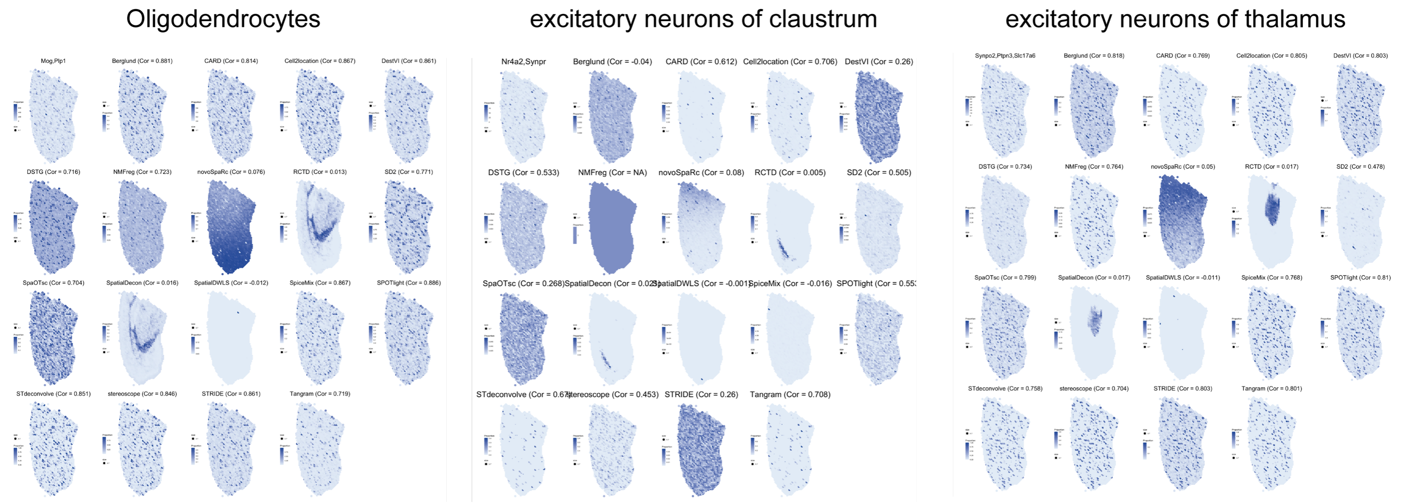

Supplementary Figure 13

The visualized prediction of three kinds of cell types (Oligodendrocytes, excitatory neurons of thalamus and excitatory neurons of claustrum) through all methods and visualized spatial expression of marker genes (ground truth). Each method is labeled by its PCC value with ground truth. Especially, NMFreg predicted the cell type called excitatory neurons of claustrum as all zero proportion which caused PCC as NaN.

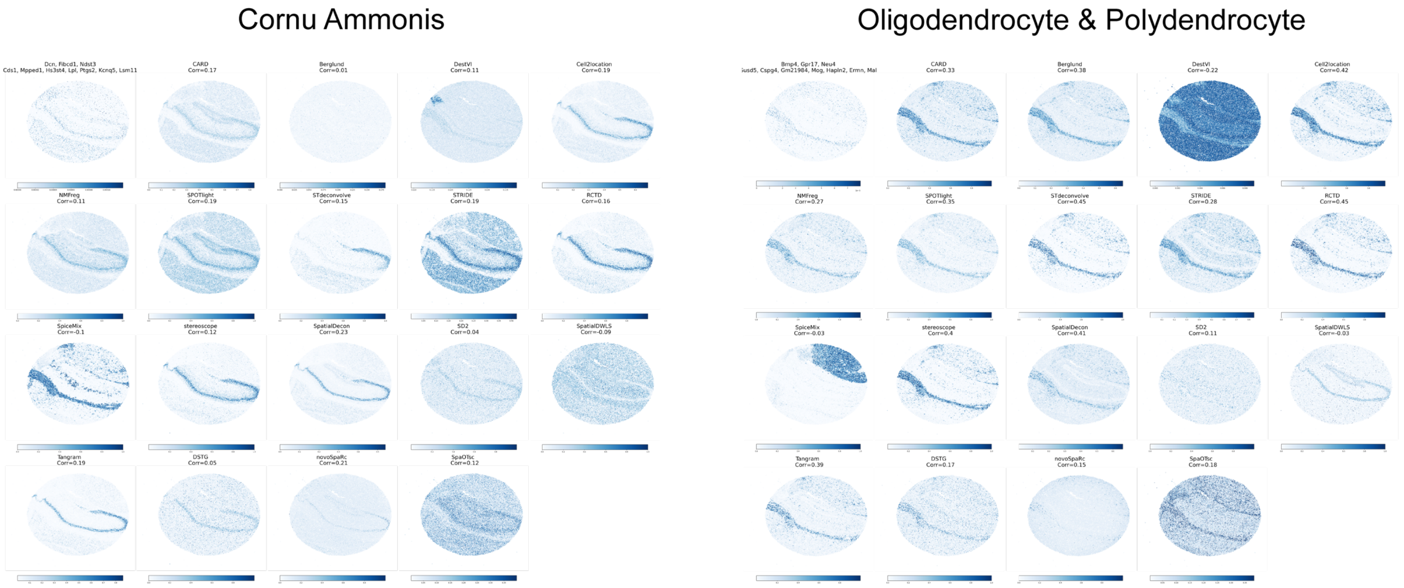

Supplementary Figure 14

The visualized prediction of two kinds of integrated cell types (Cornu Ammonis and Oligodendrocytes & Polydendrocyte) through all methods and visualized spatial expression of marker genes (ground truth). Each method is labeled by its PCC value with ground truth.

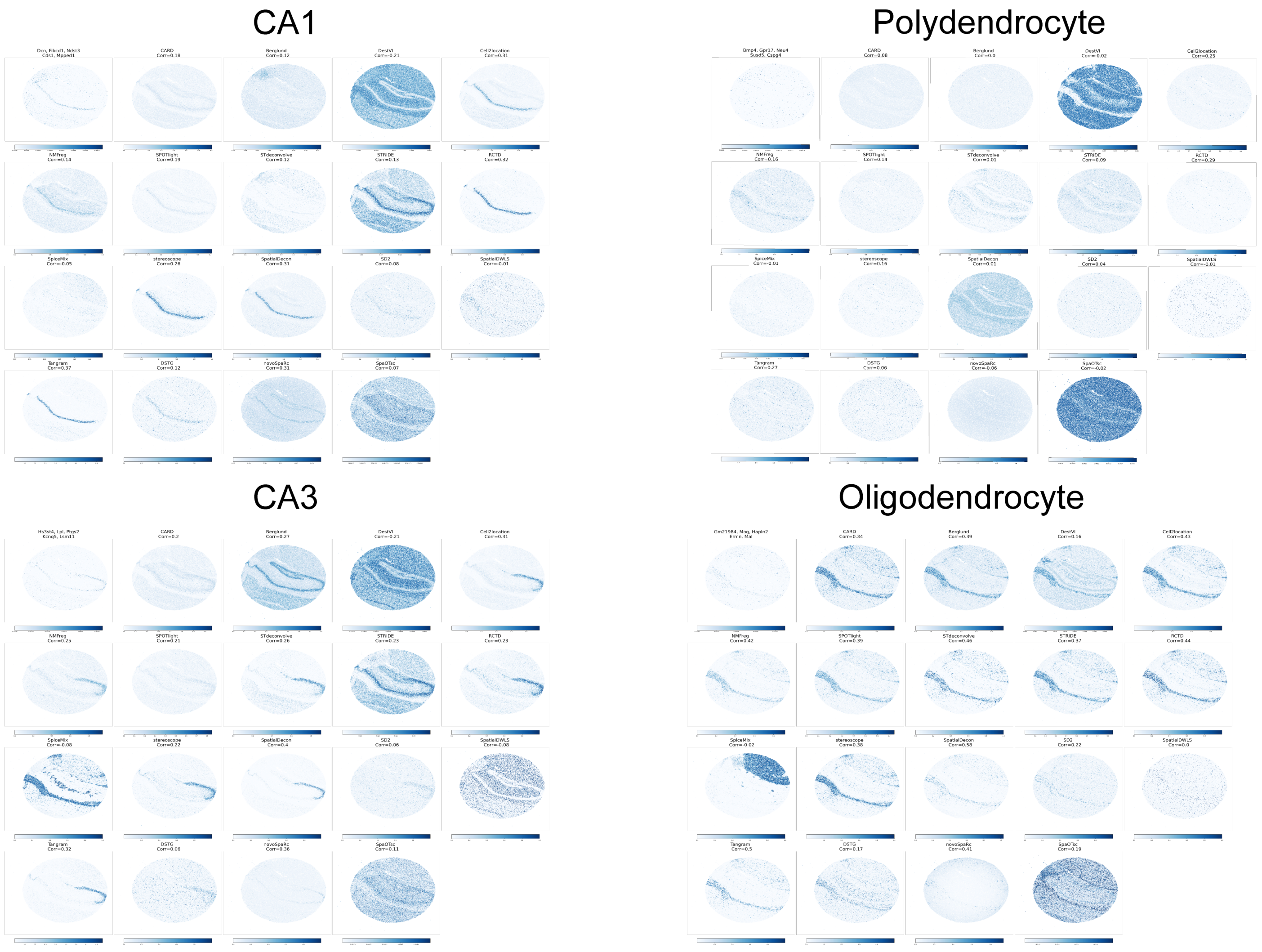

Supplementary Figure 15

The visualized prediction of four kinds of cell types (CA1, CA3, Oligodendrocytes and Polydendrocyte) through all methods and visualized spatial expression of marker genes (ground truth). Each method is labeled by its PCC value with ground truth.

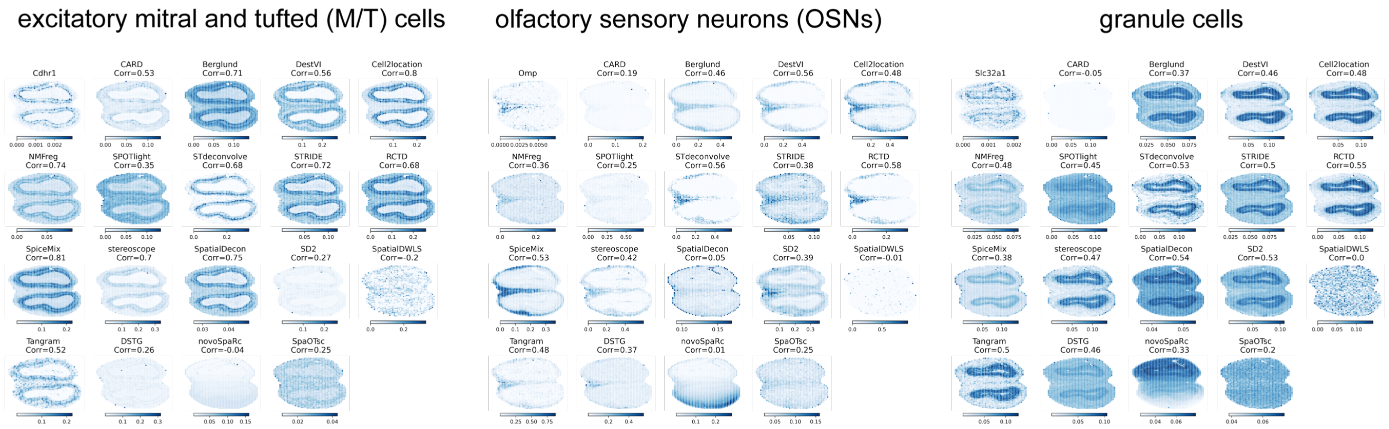

Supplementary Figure 16

The visualized prediction of three kinds of cell types (excitatory mitral and tufted (M/T) cells, olfactory sensory neurons (OSNs) and granule cells) through all methods and visualized spatial expression of marker genes (ground truth). Each method is labeled by its PCC value with ground truth.

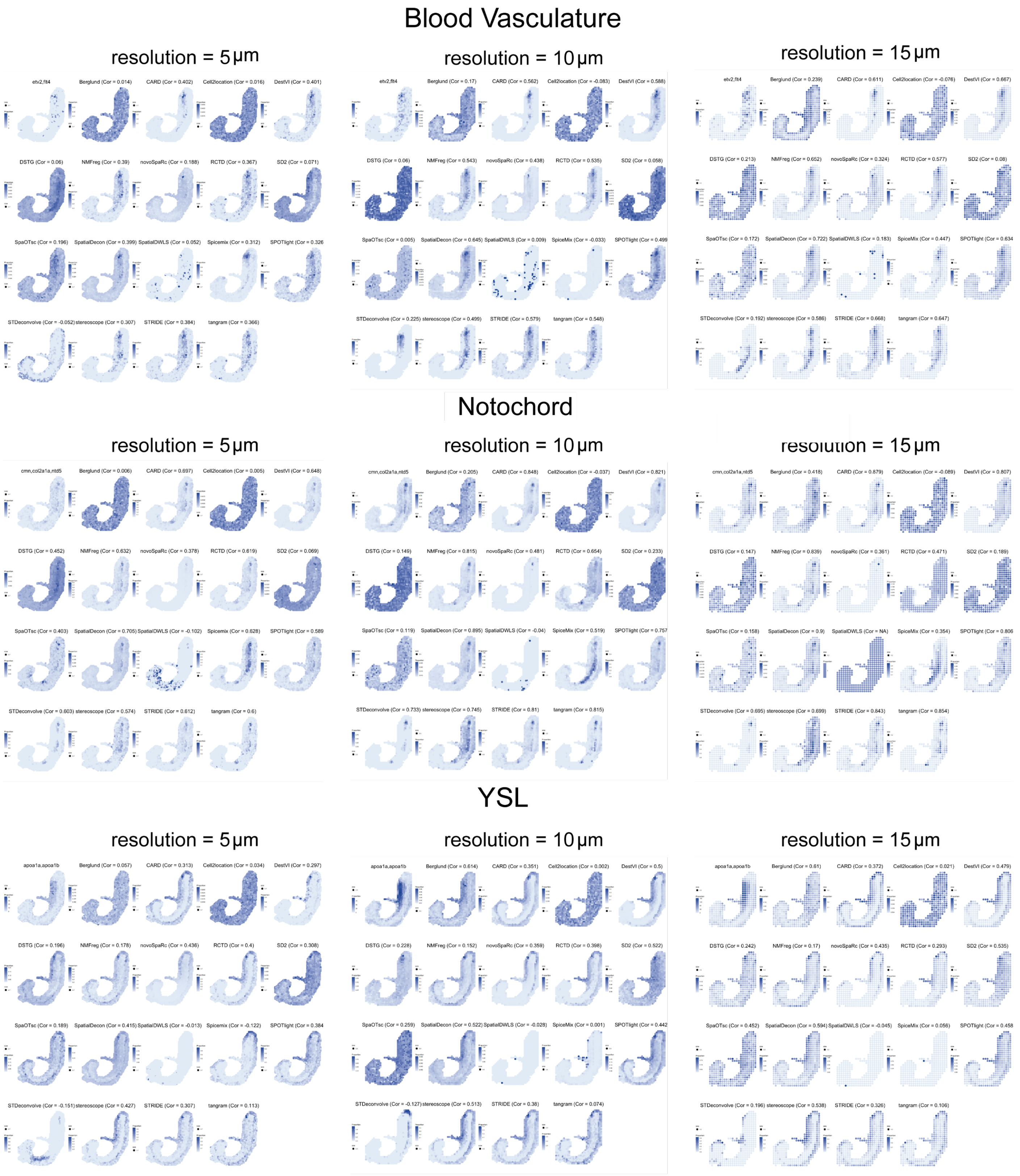

Supplementary Figure 17

The visualized prediction of three kinds of cell types (Blood vasculature, Notochord and YSL) through all methods in three kinds of resolutions (5μm, 10μm and 15μm) and visualized spatial expression of marker genes (ground truth). Each method is labeled by its PCC value with ground truth.

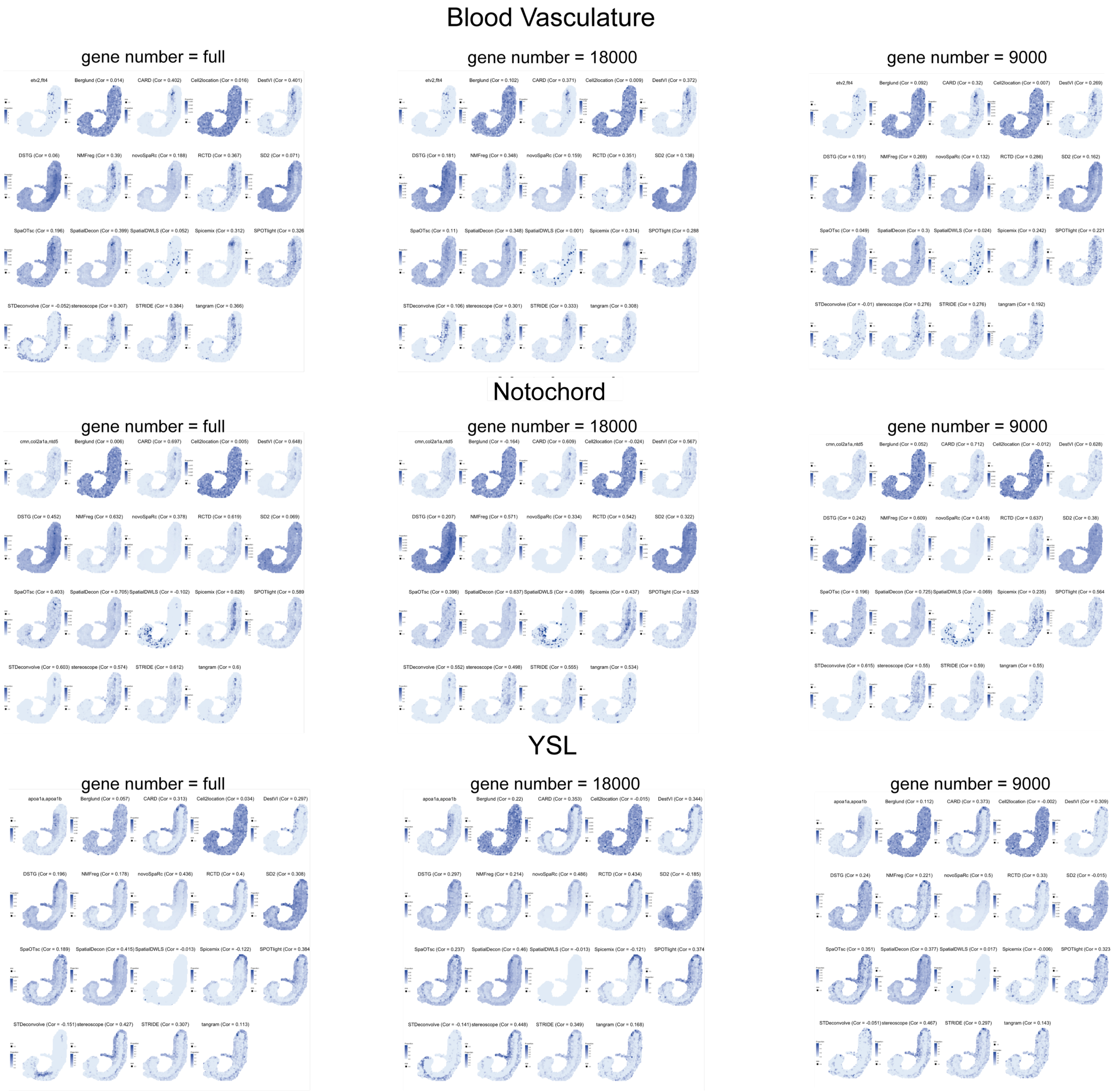

Supplementary Figure 18

The visualized prediction of three kinds of cell types (Blood vasculature, Notochord and YSL) through all methods in three kinds of gene numbers (full gene number, 18000 and 9000) and visualized spatial expression of marker genes (ground truth). Each method is labeled by its PCC value with ground truth.

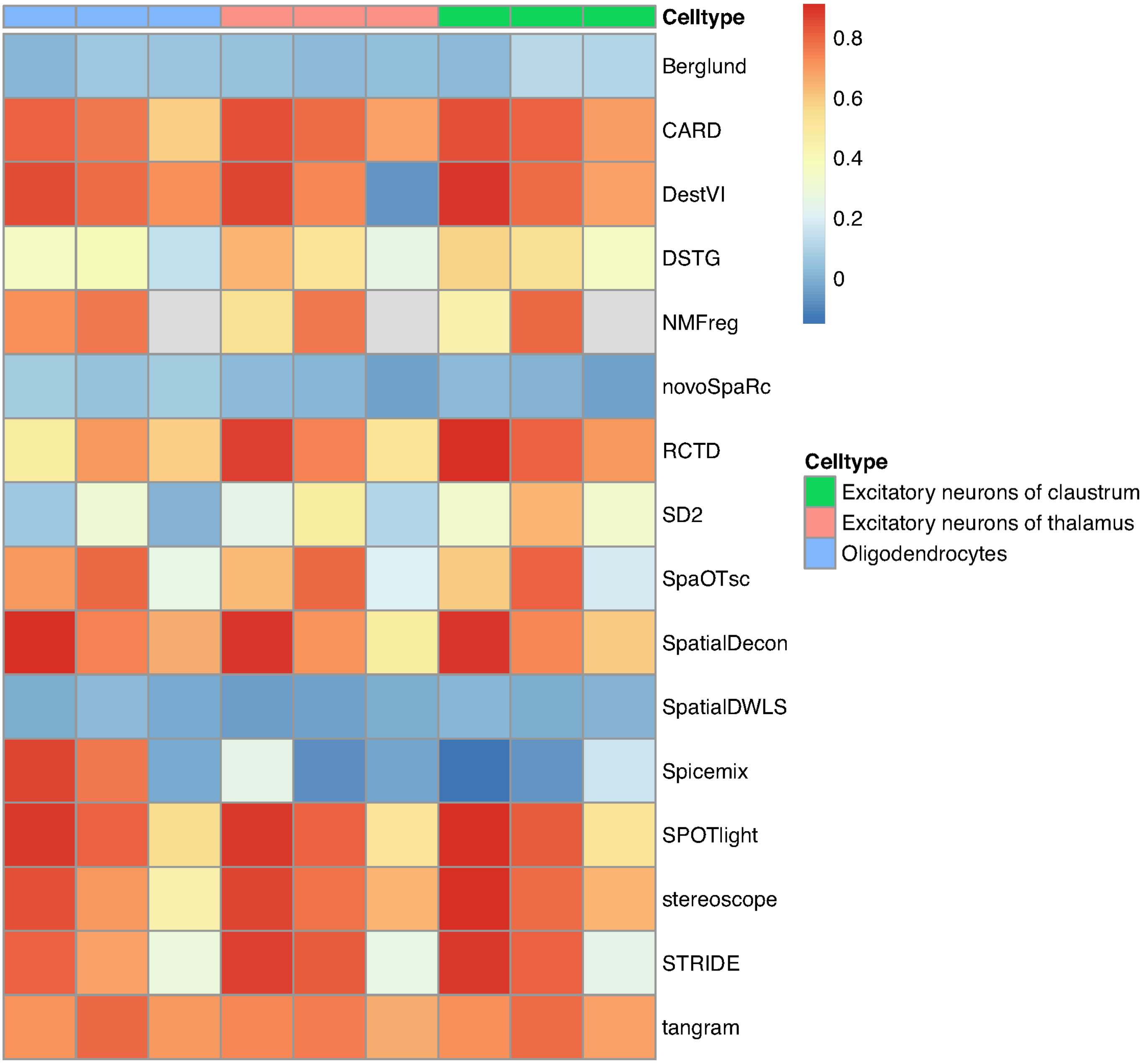

Supplementary Figure 19

The heatmap for evaluating the performance of all methods under different normalization function of spatial transcriptomics data. Three kinds of cell types are chosen to evaluate the performance. For each cell type, there are three columns representing the performance under raw count, lognorm and sctransform normalizations. Source data are provided as a Source Data file.

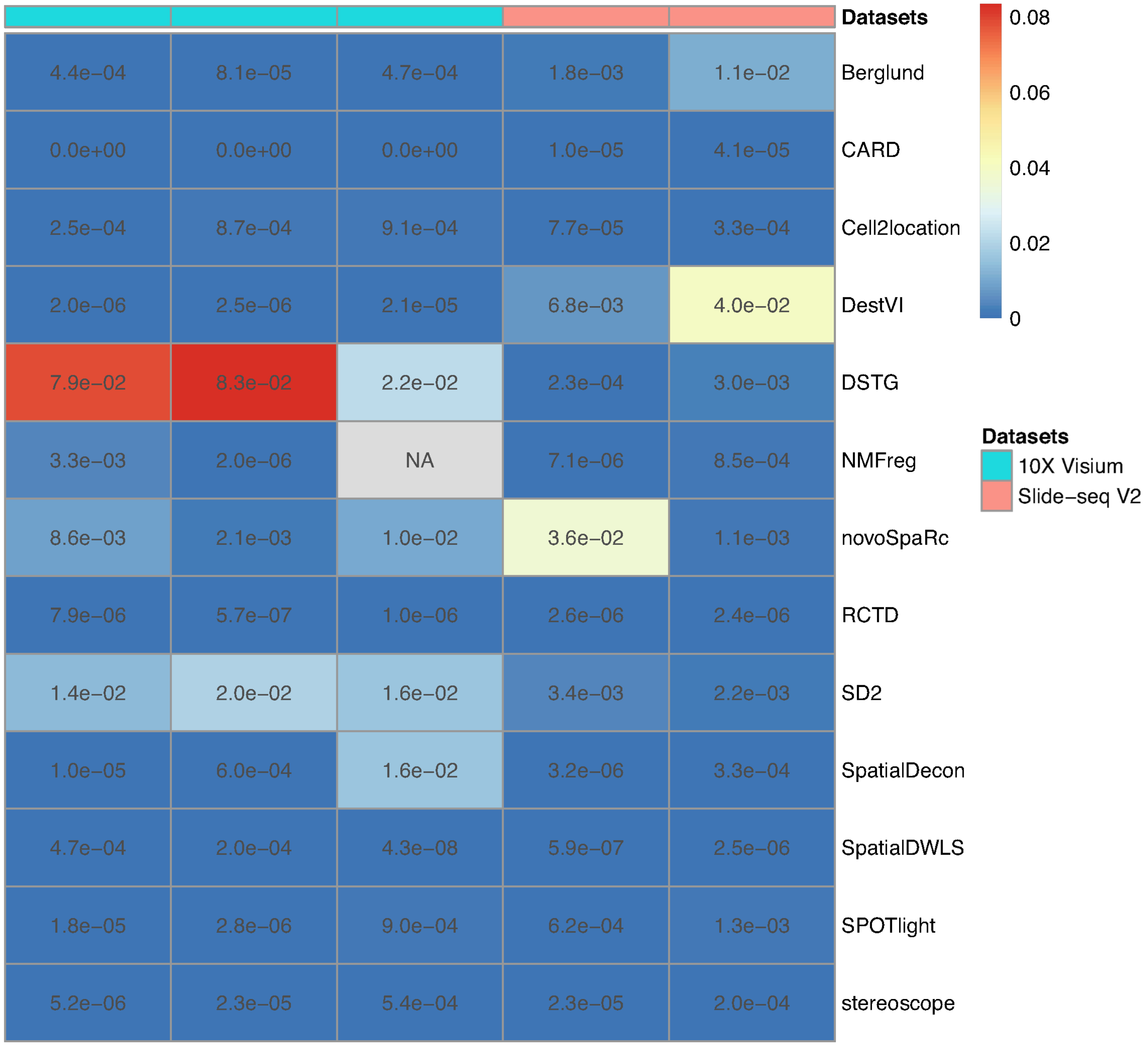

Supplementary Figure 20

The heatmap of evaluating the effects of hyperparameters of all methods. The variances of PCCs among different cell types from different datasets are measured. From the heatmap, most of methods have stable performance under different hyperparameters except DSTG which means the effects of hyperparameters do not affect the performance of all methods. For the third cell type in 10X Visium dataset, NMFreg has NA results because it outputted 0 proportions among all the spots for this cell type. Chosen hyperparamters are shown in Supp.Table 4. Source data are provided as a Source Data file.

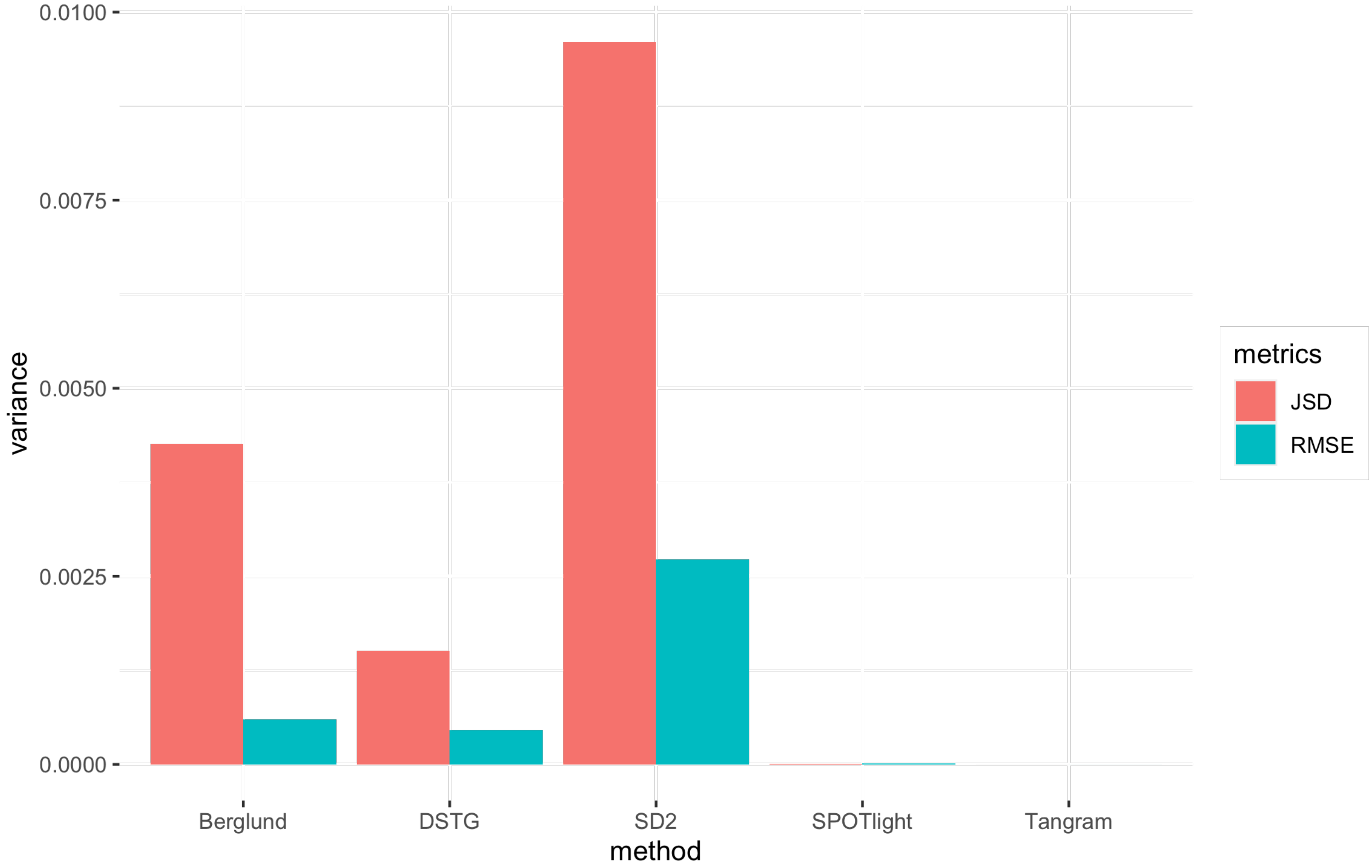

Supplementary Figure 21

The variance of JSD and RMSE among three-times repeat experiments on seqFISH+ datasets with 10000 genes per spot. Only Berglund, DSTG, SD^2, SPOTlight and Tangram show the variance and the other methods have no variance at all. Source data are provided as a Source Data file.

Supplementary Table 1
General summary of the spatial transcriptomics technologies we used in benchmarking. For MERFISH, we showed the simulated spots number with the sizes of 20 μm (50 μm, 100 μm) from each slice in MERFISH dataset. For zebrafish embryo by stereo-seq, we showed the simulated spots number with the sizes of 5 μm (10 μm, 15 μm).

Techniques	Region	cells/spots number	Gene number	Cell type number	Cells number from scRNA-seq
MERFISH	mouse brain medial pre-optic area	4504(1107,256)	135	6	1691
		4676(1102,253)			
		5066(1118,256)			
		5199(1132,256)			
		4836(1132,256)			
		4743(1131,256)			
		4495(1099,255)			
		4455(1104,255)			
		4666(1101,256)			
		4290(1111,256)			
		4349(1126,256)			
		4275(1112,256)			
seqFISH+	Cortex of mouse brain	523	10000, 6000, 3000	6	1691
ST	PDAC	316	19738	20	19736
Visium	Mouse brain	2576	31053	56	40532
Slide-seqV2	mouse hippocampus	53208	23265	11 / 17	15095
Stereo-seq	Olfactory bulb	2279	29570	18	7519
	Zebrafish embryo	2330 (660, 299)	26365,18000,9000	22	14882

Supplementary Table 2
Summary of selection of cell types and their marker genes through ST, Visium, Slide-seqV2 and two stereo-seq datasets.

Techniques	Full name of cell type	Abbreviation of cell type in dataset	Marker genes
ST	Centroacinar ductal cells	Ductal.CRISP3.high.centroacinar.like	CLDN2 [1]
	Cancer clone S100A4	Cancer.clone.A	GAPDH [1]
Visium	Oligodendrocytes	Oligo_1, Oligo_2	Mog, Plp1 [2]
	excitatory neurons of thalamus	Ext_Thal_1, Ext_Thal_2	Synpo2, Ptpn3, Slc17a6 [2]
	excitatory neurons of claustrum	Ext_Claupyr	Nr4a2, Synpr [2]
Slide-seqV2 (11 cell types)	Oligodendrocyte & Polydendrocyte	Oligodendrocyte & Polydendrocyte	Bmp4,Gpr17,Neu4,Susd5, Cspg4,Gm21984,Mog, Hapln2,Ermn,Ma [3]
	Cornu Ammonis	CA	Dcn,Fibcd1,Ndst3,Cds1,Mpped1, Hs3st4,Lpl,Ptgs2,Kcnq5,Lsm11 [3]
Slide-seqV2 (17 cell types)	CA1	CA1	Dcn,Fibcd1,Ndst3,Cds1,Mpped1 [3]
	CA3	CA3	Hs3st4,Lpl,Ptgs2,Kcnq5,Lsm11 [3]
	Oligodendrocyte	Oligodendrocyte	Gm21984,Mog,Hapln2,Ermn,Mal [3]
	Polydendrocyte	Polydendrocyte	Bmp4,Gpr17,Neu4,Susd5,Cspg4 [3]
Stereo-seq (olfactory bulb)	excitatory mitral and tufted (M/T) cells	n-15-M/TC-1, n-16-M/TC-2, n-17-M/TC-3	Cdhr1 [4]
	granule cells	n03-GC-1, n07-GC-2, n09-GC-3, n10- GC-4, n11-GC-5, n12-GC-6, n14-GC-7	Slc32a1 [4]
	olfactory sensory neurons (OSNs)	n01-OSNs	Omp [4]
Stereo-seq (zebrafish)	Notochord	Notochord	Cmn, Col2a1a, Ntd5 [5]
	YSL	Epidermis_egfl6..YSL	Apoa1a, Apoa1b [5]
	Blood Vasculature	Blood.Vessel.Endothelial.Cell	Etv2, Flt4 [5]

Supplementary Table 3

Recorded running time of all methods on MERFISH datasets with three kinds of resolutions of spots (20, 50 and 100 μm).

	MERFISH (20)	MERFISH (50)	MERFISH (100)
**Berglund**	162300.2s	7410.4s	464.1s
**NMFReg**	44.1s	11.9s	6.3s
**stereoscope**	14220.1s	7080.3s	3660.9s
**SpatialDWLS**	5714.6s	3698.8s	742.6s
**DSTG**	887.1s	245.2s	140.1s
**SPOTlight**	246.5s	79.1s	81.8s
**RCTD**	6127.2s	2996.4s	1121.1s
**Tangram**	232.5s	203.2s	193.7s
**DestVI**	1962.3s	604.9s	328.8s
**STRIDE**	98.1s	82.5s	76.8s
**SpiceMix**	91461.1s	17525.3s	4726.5s
**STdeconvolve**	1248.4s	1095.6s	580.9s
**SpatialDecon**	379.1s	87.1s	26.6s
**Cell2location**	5463.0s	2116.4s	2078.1s
**SD^2**	611.7s	301.8s	151.7s
**CARD**	536.9s	88.4s	3.9s
**novoSpaRc**	460.4s	70.7s	10.5s
**SpaOTsc**	4156s	3237s	1784s

Supplementary Table 4

Recorded running time of all methods on Slide-seq V2 datasets with three kinds of gene number (full, 16000 and 8000) and two kinds of cell-type number (11 and 17).

Gene number	Full		8000		16000	
Cell type number	17	11	17	11	17	11
Berglund	426min	426min	372min	372min	402min	402min
NMFReg	33min	31min	9min	9min	19min	19min
stereoscope	652min	654min	190min	178min	388min	373min
SpatialDWLS	78min	69min	83min	72min	78min	68min
DSTG	7min	7min	6min	6min	8min	7min
SPOTlight	13min	11min	12min	12min	14min	10min
RCTD	305min	200min	129min	85min	209min	136min
Tangram	41min	16min	14min	5min	29min	12min
DestVI	134min	123min	37min	31min	83min	72min
STRIDE	161min	86min	49min	22min	100min	59min
SpiceMix	268min	231min	145min	133min	192min	150min
STdeconvolve	129min	119min	61min	50min	81min	77min
SpatialDecon	910min	749min	537min	335min	715min	534min
Cell2location	658min	569min	465min	437min	533min	485min
SD^2	25min	24min	23min	21min	27min	25min
CARD	16min	14min	12min	11min	13min	11min
novoSpaRc	357min	339min	298min	259min	337min	320min
SpaOTsc	210min	210min	186min	186min	198min	198min

Supplementary Table 5

Chosen hyperparamters and their values for each method.

Methods	Hyperparameter	
**SPOTlight**	cl_n: 5, 10, 15	hvg:1000, 2000, 3000
**DSTG**	laerning_rate: 0.01,0.001, 0.1	epoch: 200,300,400
**SpatialDWLS**	min_gene (findMarkers_one_vs_all): 10, 20, 30	
**SD^2**	spot_num: 500, 1000, 2000,	lower_cellnum = 2, 10, 20 and upper_cellnum = 10, 20, 30
**NMFreg**	number of components: 30, 25, 35	
**Stereoscope**	number of genes: 4000， 5000，6000	st & sc epochs: 30000, 40000, 50000
**Cell2location**	max_epochs: 3000, 4000, 5000	
**RCTD**	CELL_MIN_INSTANCE: 1, 2, 3	
**SpatialDecon**	background count bg: 0.01, 0.02, 0.03	
**DestVI**	epoch for spatial model = 2000, 2500, 3000	learning_rate = 0.01, 0.001, 0.005
**CARD**	minCountGene: 5, 10, 15	
**Berglund**	Iter: 1000, 2000, 3000	
**novoSpaRc**	Alpha: 0.3,0.5,0.7	

Supplementary Table 6

During the implementation of all methods, we scored of documentary quality, code quality, installation procedures, compatibility for OS and example analysis for evaluating the usability. The range is from 1 to 5 (1: worst, 5: best).

Methods	Document quality	Code quality	Installation procedures	Compatibility for OS	Example analysis
**Berglund**	3	5	5	3	3
**NMFReg**	3	3	5	5	3
**stereoscope**	5	5	5	4	4
**SpatialDWLS**	4	2	5	4	4
**DSTG**	3	4	4	4	1
**SPOTlight**	5	5	4	4	5
**RCTD**	4	5	5	5	5
**Tangram**	3	3	5	5	5
**DestVI**	5	3	4	5	5
**STRIDE**	5	4	5	4	5
**SpiceMix**	3	3	3	4	3
**STdeconvolve**	4	3	4	4	3
**SpatialDecon**	4	4	4	5	4
**Cell2location**	4	5	5	4	4
**SD^2**	4	5	4	5	4
**CARD**	5	5	3	4	5
**SpaOTsc**	3	5	4	5	5
**novoSpaRc**	4	5	5	5	5

Supplementary References:

[1] Q. Song and J. Su, "DSTG: deconvoluting spatial transcriptomics data through graph-based artificial intelligence," *Brief. Bioinform.*, vol. 22, no. 5, p. bbaa414, Sep. 2021, doi: 10.1093/bib/bbaa414.

[2] V. Kleshchevnikov *et al.*, "Cell2location maps fine-grained cell types in spatial transcriptomics," *Nat. Biotechnol.*, 2022, doi: 10.1038/s41587-021-01139-4.

[3] D. M. Cable *et al.*, "Robust decomposition of cell type mixtures in spatial transcriptomics," *Nat. Biotechnol.*, 2021, doi: 10.1038/s41587-021-00830-w.

[4] B. Tepe *et al.*, "Single-cell RNA-seq of mouse olfactory bulb reveals cellular heterogeneity and activity-dependent molecular census of adult-born neurons," *Cell Rep.*, vol. 25, no. 10, pp. 2689–2703, 2018.

[5] C. Liu *et al.*, "Spatiotemporal mapping of gene expression landscapes and developmental trajectories during zebrafish embryogenesis," *Dev. Cell*, vol. 57, no. 10, pp. 1284–1298, 2022.
