# Supplementary material for: A comprehensive benchmarking with practical guidelines for cellular deconvolution of spatial transcriptomics
Source: Nat Commun. 2023 Mar 21;14:1548. doi: 10.1038/s41467-023-37168-7 (PMC10027878; doi:10.1038/s41467-023-37168-7)
Supplement: Supplementary file 3 — Description of Additional Supplementary Files [file 41467_2023_37168_MOESM3_ESM.pdf]

Description of Additional Supplementary Files:

Supplementary Dataset 1: The JSD score and RMSE of all methods for each cell type under the evaluation of simulated datasets.
